# Evaluation of posterior mitral isthmus ablation in the absence of a vein of Marshall

**DOI:** 10.1093/europace/euae255

**Published:** 2024-10-01

**Authors:** Clara François, Milad El Haddad, Benjamin De Becker, Maarten De Smet, Jean-Benoît Le Polain de Waroux, René Tavernier, Mattias Duytschaever, Sébastien Knecht

**Affiliations:** Dienst Cardiologie, AZ Sint Jan Brugge, Ruddershove 10, 8000 Brugge, Belgium; Dienst Cardiologie, AZ Sint Jan Brugge, Ruddershove 10, 8000 Brugge, Belgium; Dienst Cardiologie, AZ Sint Jan Brugge, Ruddershove 10, 8000 Brugge, Belgium; Dienst Cardiologie, AZ Sint Jan Brugge, Ruddershove 10, 8000 Brugge, Belgium; Dienst Cardiologie, AZ Sint Jan Brugge, Ruddershove 10, 8000 Brugge, Belgium; Dienst Cardiologie, AZ Sint Jan Brugge, Ruddershove 10, 8000 Brugge, Belgium; Dienst Cardiologie, AZ Sint Jan Brugge, Ruddershove 10, 8000 Brugge, Belgium; Dienst Cardiologie, AZ Sint Jan Brugge, Ruddershove 10, 8000 Brugge, Belgium

**Keywords:** Atrial fibrillation, Radiofrequency ablation, Mitral isthmus line, Vein of Marshall, Atrial flutter

## Abstract

**Aims:**

Achieving acute and durable mitral isthmus (MI) block remains challenging using radiofrequency (RF) catheter ablation alone. Vein of Marshall (VoM) ethanolization results in chemical damage along the MI resulting in the creation of a durable transmural lesion with a very high rate of procedural block. However, no studies have systematically assessed the efficacy of MI ablation alone when no anatomical VoM is present.

**Methods and results:**

Thirty seven patients without VoM evidenced after careful angiographic examination were included. Ablation parameters and result were compared with a matched control group in whom the posterior MI line was performed without assessing the presence of the VoM. Mitral isthmus block was achieved in 36 out of 37 patients without VoM (97%), with endocardial ablation only in 5/37 (14%) and combined endocardial and coronary sinus ablation in 32/37 patients (86%). There was a significant difference in the occurrence of block between patients without a VoM and the control group (97.3% vs. 65% respectively, *P* < 0.01), with a trend towards less needed RF {26 [interquartile range (IQR) 20–38] vs. 29 [IQR 19–40] tags [*P* = 0.8], 611 [IQR 443–805] vs. 746 [IQR 484–1193] seconds [*P* = 0.08]}.

**Conclusion:**

The absence of a VoM is associated with a very high rate of procedural block during posterior MI ablation. The higher rate of MI block in this specific population would also suggest the crucial role of the VoM (when present) in resistant MI block.

## Graphical Abstract

**Graphical Abstract:**
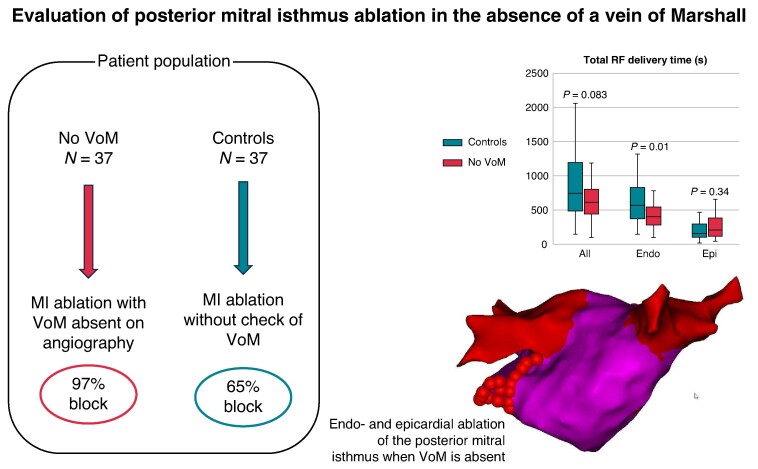

**What’s new?**

- No studies have systematically assessed the efficacy of mitral isthmus (MI) ablation alone when no anatomical vein of Marshall (VoM) is present.
- High rate of MI block (97%) is achieved with endocardial and epicardial ablation when VoM is absent. The amount of block is significantly higher compared with patients in whom the presence of the VoM was not assessed.
- There is a trend to require less radiofrequency (RF) time and fewer RF applications to block the MI as compared with a population where VoM presence has not been evaluated.

## Introduction

Linear ablation at the posterior mitral isthmus (MI) is performed to treat peri-mitral atrial tachycardia (AT), and it might increase the success rate of ablation in patients with persistent atrial fibrillation (AF) despite pulmonary vein isolation.^1–4^ However, achieving acute and durable MI block remains challenging using radiofrequency (RF) catheter ablation alone.^5–7^ This can be explained by the complex architecture of the MI, with thick muscular tissue, recesses, and epicardial connections at the level of the coronary sinus (CS) and of the Marshall bundle (MB) also called the ligament of Marshall, circumventing the endocardial MI line,^8,9^

The MB comprises multiple structures including fibrous tissue, blood vessels [vein of Marshall (VoM)], muscle bundles, ganglion cells, and autonomic nerves.^10^ It is known to be associated with multiple arrhythmias (atrial, ventricular, and accessory pathways)^11–13^ and is believed to serve as a pathway for parasympathetic and sympathetic innervation.^14,15^ Vein of Marshall ethanolization serves as a chemical ablation of the MB, and it has been shown to be a promising therapeutic target to facilitate MI block and reduce further AF incidence.^16–19^ Vein of Marshall ethanolization results in chemical damage along the MI resulting in the creation of a durable transmural lesion, with a very high rate of procedural block.^20–22^ The amount of block when performing VoM ethanolization and concomitant RF applications seems to be much higher as compared with studies where the presence of VoM was not assessed.^7,16,23^ The VoM is located on the epicardial side of the MI and cannot always be reached during endocardial applications. Therefore, when VoM ethanolization is not performed, it is most probably one of the main causes of resistant MI block, even when respecting optimized and contiguous RF ablation.^7^

However, the VoM is absent in ∼10–15% of the patients, and no study has systematically assessed the efficacy of MI RF ablation when no anatomical VoM is present. The absence of a visible VoM could result in a more difficult MI RF ablation as the epicardial side of the MI could be more difficult to reach from the endocardium. Conversely, its absence might facilitate MI block as it represents a potential cause for block failure. The purpose of this study was therefore to evaluate the procedural success rate in blocking the MI line with RF ablation in the absence of VoM.

## Methods

### Study design

This single-centre study analysed posterior MI ablation in 37 consecutive patients from AZ Sint Jan, Bruges, Belgium without the presence of a VoM demonstrated during careful angiographic examination. Patients with previous linear ablation or complex fractionated atrial electrogram ablation in the MI region were excluded. The investigated group of patients without VoM was compared with a matched control group in whom the posterior MI line was performed without assessing the presence of the VoM. The matched control patients were selected from 111 patients who underwent mitral line point-by-point RF ablation between 2018 and 2020.

### Ablation strategy

All patients from the study group and control group underwent RF catheter ablation procedures while under general anaesthesia and on either uninterrupted novel oral anticoagulation or warfarin (with International Normalised Ratio 2–3). An oesophageal temperature-monitoring probe (SensiTherm™, St. Jude Medical Inc., St Paul, MN, USA) was placed. Intravenous heparin was given after femoral venous access to achieve an activated clotting time of 300–400 s. The procedure involved introducing a decapolar CS catheter through the right femoral vein and performing double transseptal puncture using conventional long sheaths (SL0, St. Jude Medical Inc.) under transoesophageal echocardiography guidance. In addition, a Pentaray mapping catheter (Pentaray®, Biosense Webster Inc., Diamond Bar, CA, USA) and an 8F open-tip irrigated RF catheter with tip-integrated contact force (CF) sensor (Thermocool SmartTouch®, Biosense Webster Inc.) were positioned in the left atrium (LA). The CF catheter was calibrated and respiratory gating was done to create a 3D geometry and voltage map of the LA using the Carto3 System with the Pentaray catheter in sinus rhythm. All patients had pulmonary vein isolation performed before starting the MI line protocol.

### Assessment of the absence of vein of Marshall

In the study population, the CS was cannulated with a steerable sheath advanced from the right femoral vein. A sub-selector catheter with a ∼90° angle at the tip was advanced through the CS sheath with its tip pointing superiorly and posteriorly. Contrast injections through the sub-selector catheter allowed identification of the VOM while orienting the catheter tip in all the directions within the CS from its distal part of its ostium. The manoeuvre was repeated a second time when no VoM was visualized, with high-resolution fluoroscopy. Patients were included in this study when no VoM was assessed.

In the control group, no assessment of VoM was performed.

### Mitral isthmus line ablation

**Figure 1:**
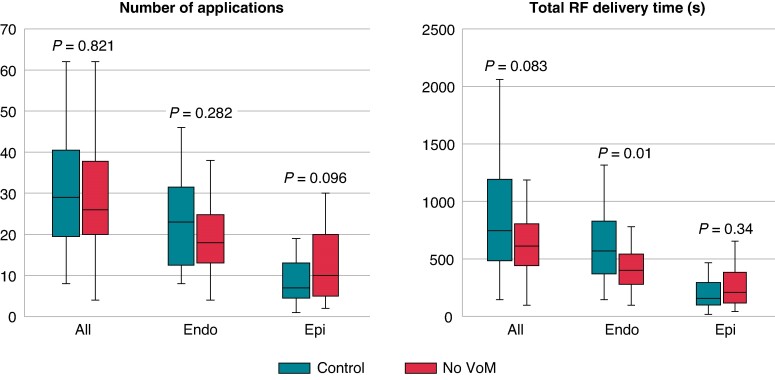
Comparison of ablation parameters between patients without VoM and control group, concerning the number of applications and total RF delivery time. RF, radiofrequency; VoM, vein of Marshall.

For all patients, RF ablation started endocardially with the aim of creating a contiguous lesion set between the mitral annulus and the south pole of the left inferior pulmonary vein (LIPV). The MI ablation involved point-by-point RF delivery (EP Shuttle ST-3077, Stockert GmbH, Freiburg, Germany) in power-controlled mode (without ramping) with a power setting of 45 W (adjusted based on catheter tip temperature feedback) and irrigation of 30 mL/min. A single line of contiguous RF applications at the MI was done, following strict criteria for contiguity (centre-to-centre inter-lesion distance ≤ 6 mm) and ablation index (AI > 550, target 600) (Hiroshi Nakagawa formula, Biosense Webster Inc.). The RF was delivered with a stable CF curve using a long steerable sheath (Agilis™ NxT steerable introducer, Abbott, IL, USA) for support. Radiofrequency applications were displayed on the anatomical map with automated tagging technology (Carto VisiTag™, Biosense Webster Inc.). If instability caused inadequate RF tagging not reaching the AI target, the RF tag was discarded and a further lesion was applied to achieve a contiguous linear set of applications with AI ≥ 550. If block was not achieved after the first endocardial lesion set, additional applications were performed epicardial from within the CS, targeting local electrograms (25–30 W, AI ≥ 350). In the CS, RF applications were performed contiguously with an inter-lesion distance of <6 mm with the RF catheter first pointing towards the LA and then towards the posterior CS musculature, resulting in circumferential ablation (*Figure 1*). In the absence of block, careful mapping was carried out to target the earliest potentials at the opposite side of the MI line from the pacing catheter.

### Assessment of bidirectional block over mitral isthmus line after ablation

Pacing manoeuvres were used as follows to assess bidirectional block across the MI line in both groups: while pacing from the LA appendage, the activation sequence in the CS is proximal to distal with complete MI line block (instead of distal to proximal if incomplete). Differential pacing from the CS proximal of the line of block was also performed while recording at the lateral side of the line. High-density activation mapping during left atrial appendage pacing was systematically performed to confirm the presence of the MI block.

### Post-procedural care and follow-up

For all patients, clinical evaluation and electrocardiogram were performed at 1, 6, and 12 months or in case of symptoms. Holter recording was performed at 6 and 12 months. Recurrence of AF and AT (>30 s) was evaluated considering a blanking period of 8 weeks. Repeat procedures were performed based on case-by-case evaluation.

### Off-line analysis of radiofrequency characteristics of mitral isthmus lines

Each procedure was exported and analysed off-line, with the determination of various parameters such as time of application (s), median power delivered (W), impedance drop (Ω), average CF (*g*), force–time integral (gs), and AI (no unit) for each RF tag. The total length of the endocardial MI was measured on the 3D navigation system as the distance between the mitral annulus 2 o'clock and the south pole of the LIPV. The MI target ablation length was measured in all patients.

### Statistical analysis

Continuous variables were expressed as mean ± SD or median [interquartile range (IQR)] for non-normal distributions, and dichotomous variables were expressed as *n* (%). The normality of the data distribution was tested by means of the Shapiro–Wilk test. All statistical analyses were performed in SPSS Statistics 24 (IBM Corp). Statistical analyses employed either parametric (Student's *t*-test) or non-parametric tests (Mann–Whitney *U* test) to assess variations in continuous clinical and ablation parameters among the groups. Categorical variables were compared using the v2 test or the Fisher's exact test, as applicable. Two-sided *P*-values were reported, and a significance level of ≤0.05 was used.

Propensity scores were calculated using binary logistic regression with the following covariates: age, body mass index, sex, left ventricular ejection fraction, and CHA_2_DS_2_VASc. Scores matching was calculated based on the nearest neighbour algorithm with a maximum distance of fifth standard deviation of pairwise distances between each patient in the ‘no VoM’ group and each patient in the control group. In total, 37 patients matched between the groups.

## Results

### Patient characteristics

There were 37 consecutive patients without demonstrated VoM (67 ± 10.5 years, 67% were male patients, LA volume 136 ± 51.1 mL) with persistent AF (81%) or perimitral flutter (11%). The median length of the ablation line was 19.5 mm (IQR 12.3–29.3). All procedures were performed under general anaesthesia, and no periprocedural complication was reported.

*Table 1* summarizes the patient characteristics from the investigated and control groups.

**Table 1:** Patient characteristics

	No VoM	Controls
Total patients	37	37
Age, years (mean ± SD)	67 ± 10.8	64 ± 7.7
Gender		
Male, *n* (%)	25 (67)	27 (73)
Female, *n* (%)	12 (33)	10 (27)
Body mass index	29.5 ± 3.5	30 ± 4.4
CHA_2_DS_2_VASc (mean)	2	2
LVEF (%)	>50	>50
LA volume (mL) (mean ± SD)	136 ± 51.1	130 ± 31.2
Arterial hypertension (%)	57	49
Diabetes mellitus (%)	19	8
Structural heart disease (%)	16	19

LA, left atrium; LVEF, left ventricular ejection fraction.

### Conduction block of mitral isthmus line and radiofrequency lesion characteristics

**Figure 2:**
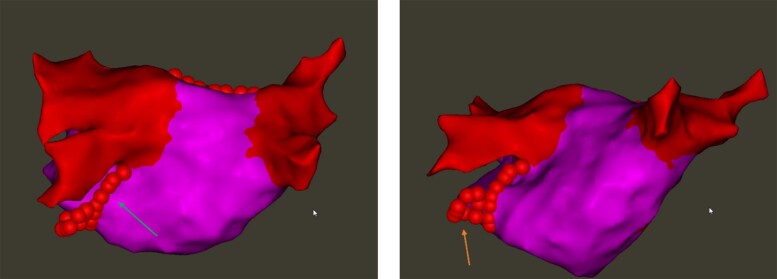
Posterior MI endocardial (left panel) and epicardial ablation (right panel) in the absence of VoM.

Mitral isthmus block was achieved in 36 out of 37 patients without VoM (97%), with endocardial ablation only in 5/37 (14%) and combined endocardial and CS ablation in 32/37 patients (86%). There was a median of 26 (IQR 20–38) RF applications at the MI line [18 (IQR 13–25) at the endocardium and 10 points (IQR 5–20) within the CS] corresponding to a median RF time of 611 s totally (IQR 443–805) [401 s (IQR 280–541) endocardially and 209 s (IQR 116–385) in the CS] (*Figure 2*).

The median impedance drop was 9 Ω (IQR 7.5–10.1) with a median CF of 19*g* (IQR 16.9–21.2). The median AI was 604 (IQR 596–606) endocardially and 406 (IQR 404.1–410.7) within the CS. The inter-tag distance was <6 mm (both endocardial and CS).

### Comparison with the control group

In 24 out of 37 patients (65%) of the control group, successful MI block was achieved through combined endocardial and epicardial ablation. There was a median of 29 (IQR 19–40) RF applications at the MI line [23 (IQR 12–31) at the endocardium and 7 (IQR 4.5–13) within the CS], corresponding to a median RF time of 746 s (IQR 484–1193) totally, 569 s (IQR 370–829) endocardially and 159 s (IQR 101–295) in the CS.

The median impedance drop was 9 Ω (IQR 8–11) with a median CF of 13*g* (IQR 17–21). Ablation index values were median 530 (IQR 510–558) at the endocardium and 405 (IQR 345–414) within the CS.

There was a significant difference in the occurrence of block between patients without a VoM and the control group (97.3% vs. 65%, respectively, *P* < 0.01), with a clear trend towards less needed RF and less applications to block the MI when no VoM is present. *Table 2* summarizes the ablation characteristics from the investigated and control groups.

**Table 2:** Ablation characteristics

	NoVom	Control	*P*-value
Block, *n* (%)	36 (97%)	24 (65%)	0.04
Number of applications (*n*)			
Endo, median (IQR)	18 (13–25)	23 (12.5–31.5)	0.3
Epi, median (IQR)	10 (5–20) 7	(4.5–13.0)	0.1
Endo + Epi, median (IQR)	26 (20–37.8)	29 (19.5–40.5)	0.8
Total RF delivery, time (s)			
Endo, median (IQR)	401 (280–541)	569 (370–829)	0.01
Epi, median (IQR)	209 (116–385)	159 (101–295)	0.3
Endo + Epi, median (IQR)	611 (443–805)	746 (484–1194)	0.08
RF duration per application (s)			
Endo, median (IQR)	22 (19.5–25.3)	27 (22–31.7)	<0.001
Epi, median (IQR)	20 (18.7–23.6)	22 (18.7–27.9)	0.2
Endo + Epi, median (IQR)	21 (19.5–23.6)	26 (22–30.4)	0.001
Ablation line length (mm)			
Endo + Epi, median (IQR)	195 (123–293)	261 (158–385)	0.1
Impedance drop (Ω)			
Endo, median (IQR)	8 (7.1–10.6)	9 (7.6–10.6)	0.7
Epi, median (IQR)	8 (7.1–11.7)	13 (7.8–17.5)	0.01
Endo + Epi, median (IQR)	9 (7.5–10.1)	9 (7.6–10.8)	0.3
Ablation index			
Endo, median (IQR)	604 (595.6–606.1)	530 (510.4–558.4)	<0.001
Epi, median (IQR)	406 (404.1–410.7)	405 (344.7–413.6)	0.3
Endo + Epi, median (IQR)	558 (454.7–603.5)	507 (451.3–531.7)	0.003
Contact force (*g*)			
Endo, median (IQR)	20.5 (18.5–23.6)	12 (10.5–17.5)	<0.001
Epi, median (IQR)	16 (13.6–18)	18 (11.7–22.8)	0.7
Endo + Epi, median (IQR)	19 (16.9–21.2)	13 (11.3–17.2)	<0.001
Inter-tag distance (mm)			
Endo, median (IQR)	5 (4.2–5.6)	6 (5.4–7.7)	<0.001
Epi, median (IQR)	6 (4.9–7.4)	7 (5.6–8.6)	0.04
Endo + Epi, median (IQR)	5 (4.6–5.8)	7 (5.8–8)	<0.001

endo, endocardial; epi, epicardial; IQR, interquartile range; RF, radiofrequency.

### Follow-up

During follow-up, 14 patients (37.8%) without VoM had recurrence of arrhythmia (AF in 8 patients, 57%, and AT in 6 patients, 43%). In five patients, a repeat procedure was performed, and in all of them, a reconnection over the MI line was observed. In all five cases, the gap in the MI line was located at the mitral annulus and posterior side of the CS.

In the control group, 18 patients (49%) had recurrence of arrhythmia (5 AF and 13 AT), with a repeat procedure in 16 patients. In 13 patients, reconnection over the posterior MI line was observed, needing additional RF applications (or VoMet) to eventually block the MI line.

## Discussion

### Main findings

Our study shows a high rate of block with linear RF ablation of the MI line when the VoM is absent. The amount of block is significantly higher than in patients in whom the presence of the VoM was not assessed.

### Posterior mitral isthmus ablation and role of the vein of Marshall

Without assessing the presence of the VoM, previous studies reported a bidirectional block at the posterior MI in around 60–80% of patients.^16,17,23^ Incomplete MI block can lead to iatrogenic ATs, often refractory to antiarrhythmic drugs and necessitating a new ablation procedure.^24^ Block failure in the MI can result from the presence of recesses, thick tissue, and epicardial connections, in particular through the MB. The MB includes muscular fibres surrounded by venous and adipose tissue and connects epicardially on both sides of the posterior MI line.^25^ Without VoM ethanolization, it may be difficult to reach by endocardial ablation and may therefore represent one of the most frequent reasons for block failure.

When VoM ethanolization is performed, it leads to an epicardial-to-endocardial elimination of the muscular continuum, thus scarring over the posterior MI area. In combination with additional endocardial and epicardial (from the CS) RF ablation, a bidirectional block can be obtained in up to 100% of patients as shown in recent studies.^26^ A study from Gillis *et al*.^17^ also showed that VoM ethanolization as a first step before RF applications results in less RF applications but a similar rate of MI block as compared with VoM ethanolization as a second step. This study also showed a higher rate of VoM visualization, suggesting that RF applications within the CS could lead to VoM alteration and precluding VoM ethanolization.

### Posterior mitral isthmus ablation without the presence of a vein of Marshall

Our study focusing on patients without VoM shows a much higher rate of procedural block and a trend to require less RF time and fewer RF applications as compared with a control group in whom the possible presence of a VoM was not assessed.

In the absence of VoM, the absence of transmurality can still occur because of thick tissue and the presence of recesses. However, it suggests that the VoM could be the main reason of procedural block failure, probably because of the distance from the endocardial side and because it is protected by adipose tissue and by the blood pool.^10,27,28^ Conversely, if the VoM is absent, deeper (AI target of 600) and contiguous (<6 mm) lesions result in a high rate of procedural MI block even if durability of the lesions was not optimal as all five patients with redo ablation had MI reconnection. However, the reconnection was located at the annulus side, which is generally not covered by VoM ethanolization.

### Clinical implications

A line connecting the mitral annulus to one of both PV circles (posterior MI line or anterior line) can be indicated in patients with a perimitral flutter or in addition to PV durable isolation in patients with persistent AF. In this case, the durability of the bidirectional block is of key importance to avoid further iatrogenic AT. When the VoM is present, VoM ethanolization should be performed given the very high rate of procedural posterior MI block and the durability of the lesion.^6,29,30^ Furthermore, chemical ablation of the VoM can have a positive impact on further AF recurrence.^16^ When the VoM is not visualized, it seems reasonable to perform the line at the posterior MI as (i) our study suggests a high rate of procedural block provided that both endocardial and epicardial ablation are performed and (ii) the MB can probably also be reached by the endocardial ablation with potential positive impact on further AF recurrence. We believe you will have a higher success rate to block the posterior MI, even in the absence of VoM, than when you perform an anterior line.

### Limitations

(i) Only a limited number of repeat procedures and re-evaluation of the MI line were performed, and therefore, no definite conclusions about the durability of MI ablation can be made with these data. (ii) A direct comparison of patients where the presence of VoM was assessed but no ethanolization occurred would give more conclusive results. However, it seemed unethical to us to not perform VoMet when a VoM was present. (iii) The ablation settings were different in the control group, with lower AI and longer inter-tag distances, which could have influenced the amount of MI block. (iv) No systematic voltage mapping was carried out before MI ablation, so no data about scarring in the MI region before ablation (and thus facilitating MI block) are available.

## Conclusions

The absence of a VoM is associated with a high rate of procedural block during posterior MI ablation. The higher rate of MI block in this specific population would also suggest the crucial role of the VoM (when present) in resistant MI block.

## Data Availability

The data underlying this article are available in the article and in its online Supplementary material.
